# The Association between Inflammation and Pulse Wave Velocity in Dyslipidemia: An Evidence-Based Review

**DOI:** 10.1155/2020/4732987

**Published:** 2020-08-18

**Authors:** Amilia Aminuddin, Md Rizman M. L. M. Lazim, Adila A. Hamid, Chua K. Hui, Mohd H. Mohd Yunus, Jaya Kumar, Azizah Ugusman

**Affiliations:** ^1^Department of Physiology, Faculty of Medicine, Universiti Kebangsaan Malaysia Medical Centre, Jalan Yaacob Latif, Bandar Tun Razak, 56000 Cheras, Kuala Lumpur, Malaysia; ^2^Department of Physiology, School of Medical Sciences, Health Campus, Universiti Sains Malaysia, 16150 Kubang Kerian, Kelantan, Malaysia

## Abstract

Dyslipidemia is associated with increased arterial stiffness (AS) which may lead to hypertension. Among the methods to assess AS are carotid-femoral and brachial-ankle pulse wave velocity. Dyslipidemia is also known to trigger inflammation. C-reactive protein (CRP) is one of the commonest inflammatory markers measured in the clinical setting. However, the association between inflammation and pulse wave velocity (PWV) in people with dyslipidemia is less studied. Therefore, this review investigated the association between inflammation (as measured by CRP) and PWV in dyslipidemia patients. The search of the literature was conducted via PubMed and Scopus database. The keywords used were “aortic stiffness” OR “arterial stiffness” OR “pulse wave velocity” OR “vascular stiffness” OR “carotid femoral pulse wave velocity” OR “pulse wave analysis” AND “inflammation” OR “c reactive protein” OR “c-reactive protein” OR “high sensitivity c reactive protein” AND “dyslipidemia” OR “hyperlipidemia” OR “hypercholesterolemia” OR “hyperlipoproteinemia” OR “hypertriglyceridemia”. The following criteria were used: (1) only full-length original articles published in English language, (2) articles that reported the association between arterial stiffness measured as carotid-femoral PWV (cfPWV) or brachial-ankle PWV (baPWV) and CRP or high-sensitivity CRP, and (3) study involving human subjects. The search identified 957 articles published between 1980 and February 2020. Only eight articles fulfilled the inclusion criteria and were used for data extraction. Five of the studies were cross-sectional studies while another three studies were interventional studies. Seven out of eight papers found a significant positive association between AS and CRP, and the correlation ranged from mild to moderate association (Pearson *r* = 0.33 to *r* = 0.624). In conclusion, inflammation is associated with increased PWV in patients with dyslipidemia. This supports the involvement of inflammation in the development of AS in dyslipidemia.

## 1. Introduction

Coronary heart disease (CHD) is the leading cause of mortality worldwide. Based on the National Health and Nutrition Examination Survey (NHANES) 2013 to 2016 data, the prevalence of cardiovascular diseases (CVD) (comprising of CHD, heart failure, stroke, and hypertension) in adults ≥ 20 years of age in the United States was 48.0% overall (121.5 million in 2016) and increases with advancing age in both males and females [1]. In 2017, the main cause of death in Malaysia was ischaemic heart diseases (IHD) with a percentage of 13.9% and this increased to 15.6% in 2018 [2]. In 2018, IHD remained as the principal causes of death for males (17.8%). For females, pneumonia remained as the principal causes of death at 12.8 percent, followed by IHD (12.2%). In 2018, IHD was also the principal cause of death in the urban areas (15.9%) and in the rural areas (15.0%) of Malaysia [2]. Dyslipidemia, which is an alteration of any lipid components in the blood, is one of the major risk factors of IHD. It was reported that the prevalence of hypercholesterolemia in Malaysia was 47.7% in 2015 [3]. Hypercholesterolemia leads to the development of atherosclerosis and CHD that can be detected by coronary angiogram or coronary calcium score (CT scan).

Among the general and hypertensive population, it was observed that lipid level has a positive association with blood pressure [4]. Studies have also found at least one lipid abnormality in most newly diagnosed hypertensive patients [5]. Such association may be due to sharing similar root of causes, or just a simple coexistence of two major CVD risk factors [6]. Another possible explanation is that dyslipidemia may induce hypertension (HTN) but the mechanism is poorly understood. Most of the available data suggest the probable positive relationship between arterial stiffness (AS) and cholesterol [7].

AS is represented as central vascular stiffness or peripheral vascular stiffness. Central vascular stiffness is also known as aortic stiffness. There are several methods to measure aortic stiffness, but the gold standard is carotid-femoral pulse wave velocity (cfPWV) [8]. cfPWV signifies the speed of the pressure waves that travel from the aorta to the femoral artery, and the unit is m/s. Measurement of aortic stiffness improves risk prediction of CVD [9]. Another marker of arterial stiffness is brachial-ankle pulse wave velocity (baPWV) which is a measure of aortic stiffness and peripheral AS [10]. baPWV is associated with other major CVD risk factors and may predict the risk of future CVD [11, 12].

Previous studies found that people with dyslipidemia had increased aortic stiffness and lipid parameters had a significant association with aortic distensibility. A study by Vallée et al. [13] found that non-high-density lipoprotein cholesterol (HDL-C), total cholesterol (TC), and TC/HDL-C were significantly associated with aortic stiffness. It was found that arterial stiffness increases in dyslipidemia. Besides, dyslipidemia was associated with higher pulse wave velocity (PWV) in young subjects and the elderly [14, 15].

Increased AS may be related to increased inflammation [16]. Dyslipidemia causes increased inflammation which may act as a mechanism towards increased AS [17]. However, previous studies are far from conclusive and interpretation is hampered by the use of multiple different techniques, indices, and small sample sizes. Hence, the objective of this review is to identify relevant studies focusing on the association between inflammation and PWV in people with dyslipidemia. Unravelling the link between inflammation and PWV in dyslipidemia will lead to a better understanding on the mechanism of AS and HTN in dyslipidemia patients. In this review, we focused on the commonest inflammatory markers that are measured clinically which are C-reactive protein (CRP) and high-sensitivity CRP (hs-CRP).

## 2. Materials and Methods

This study is aimed at identifying previous studies that investigated the association between PWV and inflammation in people with dyslipidemia. The literature search was conducted between November 2019 and February 2020 from two online databases, namely, PubMed and Scopus. The following keywords were used as search strategy: (“aortic stiffness”) OR (“arterial stiffness”) OR (“pulse wave velocity”) OR (“vascular stiffness”) OR (“carotid femoral pulse wave velocity”) OR (“pulse wave analysis”) AND (“inflammation”) OR (“c reactive protein”) OR (“c-reactive protein”) OR (“high sensitivity c reactive protein”) AND (“dyslipidemia”) OR (“hyperlipidemia”) OR (“hypercholesterolemia”) OR (“hyperlipoproteinemia”) OR (“hypertriglyceridemia”).

### 2.1. Study Inclusion and Exclusion Criteria

The articles that were retrieved from the database following the keywords were reviewed independently by two authors (A.A. and A.U.) based on the following criteria: (1) only full-length original articles published in English language, (2) articles that reported the association between aortic stiffness measured as carotid-femoral pulse wave velocity (cfPWV) or brachial-ankle pulse wave velocity (baPWV) and C-reactive protein (CRP) or high-sensitivity CRP, and (3) study involving human subjects.

### 2.2. Article Screening

In this study, the screening of the articles was conducted in three steps. Firstly, the articles were excluded based solely on the title. Secondly, the articles that were not relevant to the association between arterial stiffness and CRP among dyslipidemia subjects were excluded by reading through the abstracts. Finally, the remaining articles that did not match the inclusion criteria were excluded by reading the full text thoroughly. The study design, age and sex of the subjects, types of treatment, method of measurement, results, and references of each study were recorded.

## 3. Results

**Figure 1:**
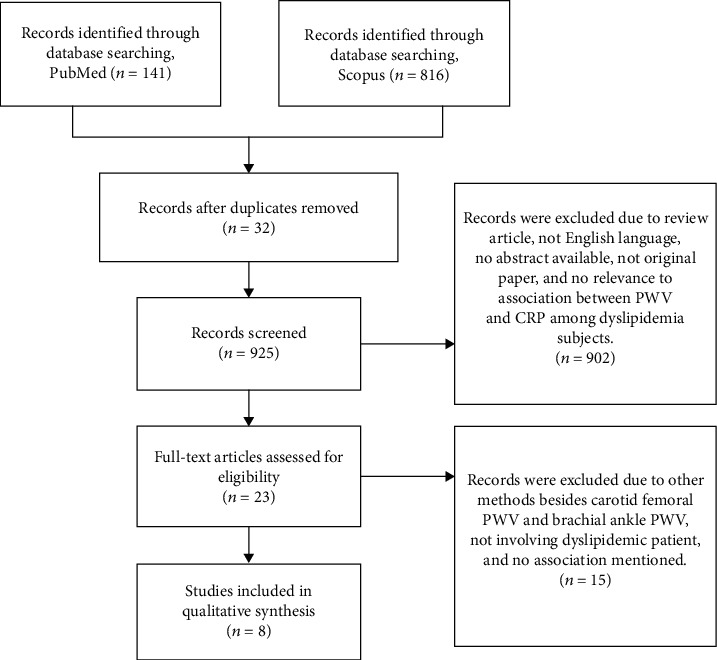
Flowchart of selection of the related articles.

A total of 957 articles were retrieved from two online databases, of which 141 articles were from PubMed and 816 articles were from Scopus. The articles were published between 1980 and February 2020. Thirty-two articles were removed due to duplication. After reviewing the titles and abstracts, 902 articles were excluded. The full-length articles for the remaining articles (23 articles) were obtained and were reviewed thoroughly. From these 23 articles, only eight studies were included in this review. The process of article selection is shown in Figure 1.

Table 1 summarised all the eight studies that were included in this review. The details of all the parameters' value are included in Table 2. From eight studies, four studies measured AS using cfPWV [18–21] while another four studies measured baPWV [22–25]. Five studies were cross-sectional studies [18, 20, 21, 24, 25], and the other three were interventional studies [19, 22, 23]. For the interventional studies, two trials involved drug treatment while another one involved dietary intervention. Measurement of PWV was conducted before and after the intervention. The studies mostly involved middle-aged and older subjects with dyslipidemia. Their mean BP were within the normal value (SBP range between 114 mmHg and 136 mmHg and DBP range: 61-78 mmHg) (Table 2).

In terms of whether the subjects were on drug therapy or not, from total eight studies, three studies involved subjects on drug treatment [18, 22, 23], one study involved diet modification [19], two studies involved subjects with and without medications [21, 25], and another two studies were not taking any medication [20, 24] (Table 1). None of the subjects was taking anti-inflammatory agents.

Most of the studies found that hs-CRP correlated with arterial stiffness as measured by either cfPWV or baPWV. For a cross-sectional study, studies by Scicali et al. [18], Pirro et al. [20], Cheng et al. [24], and Wang et al. [25] found that hs-CRP was positively correlated with PWV in dyslipidemia patients. The association ranged from mild (Pearson *r* ranged between 0.1 and 0.3) to moderate (Pearson *r*/Spearman's rho ranged between 0.4 and 0.6) association. Only one study did not observe a significant association between aortic stiffness and CRP [21]. For the interventional study, Pirro et al. [19] showed that eight weeks low-fat diet was able to reduce baPWV and this reduction was associated with a reduction in plasma CRP (*r* = 0.59, *β* = 0.38, *P* < 0.05 for both). In another study, Hongo et al. [22] found that among dyslipidemia subjects who had no history of coronary artery disease (CAD) or stroke but had more than three major cardiovascular risk factors, treatment with either rosuvastatin or fluvastatin for 12 months was able to reduce baPWV. This reduction was significantly associated with a reduction in hs-CRP (*r* = 0.36 and *r* = 0.33, *P* < 0.05 for both). A similar author also found that treatment of patients with CAD and dyslipidemia with fluvastatin for five years reduced baPWV. The reduction in baPWV correlated with a reduction in hs-CRP (*r* = 0.49, *P* < 0.001) [23]. From eight studies, only one study had further analyzed the effects of a combination of dyslipidemia and increased inflammation and dyslipidemia without increased inflammation towards aortic stiffness [24] (data not shown). They found that those with dyslipidemia and increased inflammation had significantly higher AS when compared to dyslipidemia without increased inflammation.

From eight studies, only seven studies reported both the associations between PWV and CRP and PWV and lipid level [19–25]. From seven studies, three studies found that both CRP and lipid were associated with PWV [20, 24, 25]. From these three studies, two studies found that the association between PWV and CRP were stronger than PWV and lipid level [24, 25].

We also look into the difference in brachial BP between the subjects with dyslipidemia and the healthy controls in the studies included. There were four studies that can be used for the BP comparison since the studies also involved healthy controls. All the four studies observed no difference in brachial BP between the groups [19–21, 24].

## 4. Discussion

This review found that most of the studies observed that inflammation is associated with increased PWV in dyslipidemia. This might explain the role of inflammation in inducing AS among the dyslipidemia patients which may lead to hypertension. The arterial wall contains collagen fibres that provide tensile strength against the high pressure from the blood ejected by the heart, as well as elastin fibres that give the wall elastic behaviour to accommodate the blood volume. During systole, a greater volume of blood from the heart enters the arteries than flowing into the arterioles since arterioles have higher resistance compared to arteries. In order to store the excessive blood volume, the arteries expand due to its elasticity. During diastole, the stretched arterial wall passively recoils and exerts pressure to the blood. This pushes the blood downstream and provides continuous blood flow to the organ during diastole [26]. Damage to the collagen and elastin fibres leads to arterial stiffness whereby the artery losses its elasticity. This leads to an increase in systolic and diastolic blood pressure and pulsatile afterload which promote left ventricular remodeling, dysfunction, and failure [27].

In this review, we found that there was no difference in terms of brachial blood pressure between subjects with dyslipidemia and the healthy controls. We were not sure why this occurs since previous studies suggest a positive correlation between dyslipidemia and increased BP [28]. The values of aortic stiffness were also increased in those studies (except study by Cheng et al. [24]) which suggest that there was already vascular dysfunction. One mechanism that may be involved is that dyslipidemia may compromise the central artery first (as evidence by increased aortic stiffness) compared to the peripheral artery. This may lead to a change in aortic blood pressure [29] and spared the brachial BP. The subjects involved may be at the early phase of dyslipidemia since they were newly diagnosed and the majority of the cases were still not on medication.

Previous studies found that dyslipidemia was closely related to inflammation. For example, patients with dyslipidemia had a higher level of inflammatory markers compared to normal controls [30–33]. In diabetic patients, inflammatory markers were significantly associated with lipid parameters [34]. Inflammation was also associated with dietary cholesterol intake as observed in 8105 individuals without CVD. In the study, dietary food and beverage history was obtained via 24 h diet recall [35]. Another study involving 17689 participants also showed that hs-CRP concentration was modulated by dietary fatty acid intake [36]. Meanwhile, treatment with simvastatin was able to reduce the LDL-C level and subclinical inflammation [37].

There are several mechanisms that link dyslipidemia and inflammation. LDL-C and inflammation are involved in the pathogenesis of atherosclerosis. LDL-C enters the vascular intimal layer and triggers inflammation once it becomes oxidized. The induction of proinflammatory conditions is mediated via lectin-like oxidized LDL-C receptor-1 (LOX-1) [38]. This leads to increased expression of adhesion molecules such as vascular cell adhesion molecules 1 (VCAM-1), intercellular adhesion molecule 1 (ICAM-1), chemokines, and growth factors such as macrophage colony-stimulating factors (M-CSF) from the endothelial cells and vascular smooth muscle cells that attract monocytes and inflammatory cells to the area. Monocytes differentiate into macrophage and induce further inflammation via the secretion of interleukin- (IL-) 1*β*, tumour necrosis factor-alpha (TNF-*α*), IL-1, IL-6, IL-12, IL-15, and IL-18 [39]. The role of inflammation in the pathogenesis of atherosclerosis has been reviewed by several authors recently [40, 41].

The involvement of inflammation in AS had been shown in previous studies [16, 42, 43]. This can be divided into acute effect (alteration in function) and chronic effect (alteration in structure) [16]. Acute effect of inflammation towards AS is mainly due to alteration in nitric oxide (NO) bioavailability and NO production by endothelial cells. NO is produced from L-arginine and oxygen by the action of endothelial NO synthase (eNOS) and tetrahydrobiopterin (BH_4_) as the cofactor [44]. Inflammatory cytokines decrease the half-life of eNOS mRNA [45] and inhibit eNOS activation [46]. The inflammatory cytokines also reduce L-arginine by inducing the formation of arginase and superoxides that subsequently oxidize BH_4_ and bind to NO to form peroxynitrite [16]. In a human study, influenza vaccination induced inflammation which led to endothelial dysfunction and increased AS [47]. All these events lead to endothelial dysfunction and increased AS.

Inflammation also leads to AS by changing the structure of the arterial wall. There are several mechanisms that contribute to this change. A study found that CRP induced leucocytes to release matrix metalloproteinases that degraded the elastin fibres in the arterial wall [48, 49]. Leucocyte accumulation in response to the proinflammatory cytokines also activates vascular smooth muscle cell migration, proliferation, and secretion of various mediators such as endothelin, angiotensin II, proteases, collagen, and proteoglycans that regulate vascular contractility which lead to increased stiffness [50]. Inflammatory mediators such as CRP induce vascular smooth muscle cells to produce bioapatite that promotes arterial wall calcification [51–53]. Inflammation also alters the cellular composition of the extracellular matrix (ECM). In intermediate and advanced atherosclerotic lesions, there are upregulations of glycosaminoglycans, decorin, versican, biglycan, and hyaluronan [54]. Increased hyaluronan leads to water trapping, swelling, and formation of viscous gel that allows ECM to resist compression forces [54].

Based on a limited number of studies, we found that the association between PWV and CRP was stronger than the associations between PWV and lipid level. It is still early to conclude this; however, based on the above information, this happens maybe because the effects of inflammation are more direct (acute effect) compare to the effects of lipid parameter towards AS. The effects of dyslipidemia towards AS is mediated by inflammation.

A study by Cheng et al. found that the cholesterol year score (CYS) was a significant determinant of hs-CRP [24]. CYS was determined by the duration and severity of hypercholesterolemia. Both CYS and hs-CRP were also predictors for increased baPWV. Further analysis found there was a synergistic effect of dyslipidemia and increased inflammation towards aortic stiffness [24]. Increased inflammation was defined as CRP > 1 mg/dL. Thus, it is suggested that the measurement of CRP is beneficial as a guide for management, and the use of an anti-inflammatory agent as a complementary treatment of antihyperlipidemia in reducing aortic stiffness.

### 4.1. Study Limitation

This review is based on a limited number of articles published in English language only. Besides, only CRP and hs-CRP are taken as the inflammatory markers assessed. There are other inflammatory mediators that can be assessed such as IL-6 and TNF-*α*. On the other hand, there are other available methods to assess arterial stiffness such as augmentation index and finger photoplethysmography fitness index (PPGF) [55–57]. However, in this review, we only focus on cfPWV and baPWV since both methods have been found to be associated with future CVD as mentioned earlier [9, 12]. The subjects also involved those who were on various medications which may affect the results. Lastly, we did not look into the different types of lipid components that may have different effects on AS.

## 5. Conclusions

Inflammation is associated with increased pulse wave velocity in dyslipidemia patients. This suggests the involvement of inflammation in the development of arterial stiffness in dyslipidemia.

## Figures and Tables

**Table 1:** Previous studies focusing on the association between PWV and CRP in people with dyslipidemia.

Ref.	Study design & subject characteristic	Mean age (years)	Male subjects (%)	Correlation		Methods		BP comparison between control and dyslipidemia subjects
				PWV & CRP	PWV & lipid	cfPWV/baPWV	CRP/hs-CRP	
[18]	Cross-sectional study.					cfPWV = SphygmoCor CVMS.	hs-CRP = enzymatic method.	Both groups had dyslipidemia and BP could not be compared.
	39 subjects with a genetically confirmed diagnosis of FH. On statin for 3.5 (2.5–7.5) years.	48.87 ± 12.29	53.8	*β* = 0.245, *P* < 0.05.	—			
	39 dyslipidemic subjects without a clinical diagnosis of FH. On statin for 2 (1.5–3) years.	50.57 ± 11.16	51.3	—	—			
[19]	Interventional study. 35 subjects with primary hypercholesterolemia. Subjects received 8-week low-cholesterol/low saturated fat diet (30% total fat, 5% saturated fat, cholesterol < 200 mg/daily).	58 ± 14	—	Δ in AS was associated with Δ of CRP (*r* = 0.59, *β* = 0.38, *P* < 0.05 for both).	Δ in PWV was not associated with Δ in TC and LDL-C (*P* > 0.05).	cfPWV = SphygmoCor Vx system.	CRP = latex-enhanced CRP assay.	No difference in brachial SBP and DBP between control and dyslipidemia subjects at baseline.
	15 normocholesterolaemic subjects (baseline control).	57 ± 13	—	—	—			
[20]	Cross-sectional study.					cfPWV = SphygmoCor Vx system.	CRP = latex-enhanced CRP.	No difference in brachial SBP and DBP between control and dyslipidemia subjects.
	HC subjects not on tx (*n* = 60).	57 ± 14	47	*r* = 0.55, *P* < 0.001. Standardized *B* = 0.3, *P* = 0.03.	LDL-C was IV for PWV (model 2) (standardized *B* = 0.31, *P* = 0.01)			
	Normocholesterolaemic controls (*n* = 25).	57 ± 13	44	—	—			
[21]	Cross-sectional study.					cfPWV = Complior®.	CRP = immunonephelometry.	No difference in brachial SBP and DBP between FH and normolipidemic subjects.
	FH (*n* = 89) 31.5% on treatment	39 ± 14	38.2	No association	PWV was associated with TG (*r* = 0.24, *P* = 0.03).			
	Normolipidemia (*n* = 31)	40 ± 12	51.6	—	—			
[22]	Drug intervention. Subjects with dyslipidemia and more than 3 major cardiovascular risk factors but no history of CAD or stroke. Followed up for 12 months (protocol 1).					baPWV = pulse pressure analyzer.	hs-CRP = standard method.	Both groups had dyslipidemia, and BP could not be compared.
	Group A (*n* = 38): received 2.5-5 mg/day of rosuvastatin.	63.5 ± 11.7	47.4	Δ in baPWV was associated with Δ in hs-CRP (*r* = 0.36, *P* < 0.05).	No assc.			
	Group B (*n* = 37): received 20-40 mg/day of fluvastatin.	62.8 ± 10.4	48.6	Δ in baPWV was associated with Δ in hs-CRP (*r* = 0.33, *P* < 0.05).	No assc.			
[23]	Intervention (protocol 1) Patients with CAD and dyslipidemia were given drug treatment and were followed for 5 years.					baPWV = pulse pressure analyzer.	hs-CRP = standard method.	Both groups had dyslipidemia, and BP could not be compared.
	Group A: *n* = 50, received 20–40 mg/day of Fluvastatin. 44% HPT, 28% DM.	69.3 ± 9.5	42	Δ in baPWV correlated significantly with Δ in CRP level (*r* = 0.49, *P* < 0.001) after 5 years tx.	No assc.			
	Group B: *n* = 43, received 200–400 mg/day of bezafibrate. 53% HPT, DM 35%.	69.6 ± 9.4	41.9	No assc.	No assc.			
[24]	Cross-sectional study.					baPWV = using a novel device	hs-CRP = latex-based immunoassay	No difference in brachial SBP and DBP between control and dyslipidemia subjects.
	FH (*n* = 35) not on tx.	37.1 ± 17.8	51·4	Spearman's rho = 0.414, *P* = 0.013 CRP is an IV for baPWV (standardized *B* = 0.303, *P* < 0.05).	TC (Spearman's rho = 0.354, *P* < 0.05), LDL-C (0.358, *P* < 0.05)			
	17 healthy control.	33.0 ± 15.0	47					
[25]	Cross-sectional study. 153 dyslipidemic subjects (aged 26-68 years) selected from medical screening. No DM. Men = 34 (40.1%) on tx. Women = 31 (45%) on tx.	Men: 42.4 ± 1.04 Women: 42.5 ± 1.3	54.9%	(*r* = 0.624, *P* < 0.0001) CRP is an IV for baPWV (standardized *B* = 0.444, *P* < 0.0001).	TC (*r* = 0.518, *P* < 0.0001) and HDL-C (*r* = −0.313, *P* < 0.0001). No assc. with TG and LDL-C.	baPWV = automated device (VP-1000; Colin Corp., Komaki, Japan)	hs-CRP = latex-enhanced immunoturbidimetric assay	All subjects had dyslipidemia, and BP could not be compared.

Abbreviations: Assc: association; baPWV: brachial-ankle pulse wave velocity; BP: blood pressure; CAD: coronary artery disease; CRP: C-reactive protein; DBP: diastolic blood pressure; DM: diabetes mellitus; FH: familial hypercholesterolemia; HC: hypercholesterolaemic; HPT: hypertension; hs-CRP: high-sensitivity C-reactive protein; IV: independent variable; LDL-C: low-density lipoprotein cholesterol; PWV: pulse wave velocity; SBP: systolic blood pressure; tx: treatment; Δ: changes.

**Table 2:** Values of related parameters in each study.

Ref.	Subjects		CRP/hs-CRP value	PWV value	SBP/DBP (mmHg)	HR (bpm)	TC	HDL-C	LDL-C	TG
[18]	FH		0.10 (0.05–0.16) mg/dL	8.63 ± 0.92 m/s	114.7 ± 12.31/69.9 ± 10.21	nm	209.77 ± 13.20 mg/dL	52.79 ± 10.8 mg/dL	126.75 ± 12.05 mg/dL	92 (74.5–110.5) mg/dL
	Non-FH		0.08 (0.04–0.14) mg/dL	6.68 ± 0.73 m/s	115.8 ± 10.67/71.36 ± 9.17	nm	206.14 ± 13.14 mg/dL	55.77 ± 12.14 mg/dL	124.36 ± 11.08 mg/dL	106 (82.75–129.5) mg/dL
	FH vs. non-FH (*P* value)		ns	<0.05	ns	—	ns	ns	ns	ns

[19]	Before diet intervention		1.8 (0.8–2.7) mg/L	8.9 ± 2.0 m/s	125 ± 9/78 ± 10	64	6.5 ± 0.8 mmol/L	1.3 ± 0.3 mmol/L	4.5 ± 0.7 mmol/L	1.3 ± 0.5 mmol/L
	After diet intervention		1.1 (0.4–1.7) mg/L	8.1 ± 1.9 m/s	123 ± 10/74 ± 13	67 ± 10	6.2 ± 0.6 mmol/L	1.3 ± 0.3 mmol/L	4.3 ± 0.5 mmol/L	1.2 ± 0.5 mmol/L
	Before vs. after intervention (*P* value)		0.001	0.02	ns	ns	0.008	ns	0.02	ns

[20]	HC subjects		1.65 (0.7–2.9) mg/L	8.8 ± 2.3 m/s	131 ± 10/78 ± 10	62 ± 9	6.4 ± 0.8 mmol/L	1.3 ± 0.3 mmol/L	4.5 ± 0.7 mmol/L	1.2 ± 0.5 mmol/L
	Control subjects		0.70 (0.3–1.7) mg/L	6.7 ± 1.3 m/s	128 ± 7/73 ± 12	60 ± 12	5.2 ± 0.8 mmol/L	1.3 ± 0.3 mmol/L	3.3 ± 0.6 mmol/L	1.1 ± 0.7 mmol/L
	HC vs. control (*P* value)		0.03	<0.001	ns	0.4	<0.001	0.8	<0.001	0.3

[21]	FH		1.7 (0.2–34) mg/L	9.2 ± 1.5 m/s	121 ± 14/77 ± 9	nm	359 ± 97 mg/dL	50 ± 13 mg/dL	279 ± 97 mg/dL	133 ± 58 mg/dL
	NL		1.3 (0.2–8) mg/L	8.5 ± 1 m/s	117 ± 8/76 ± 6	nm	174 ± 27 mg/dL	55 ± 13 mg/dL	102 ± 26 mg/dL	83 ± 36 mg/dL
	FH vs. NL (*P* value)		ns	0.007	ns		<0.001	ns	<0.001	<0.001

[22]	Group A	Before tx	2.86 (1.87-6.87) mg/L	1889 ± 341 cm/s	135 ± 17/61 ± 8	70 ± 11	nm	43 ± 5 mg/dL	146 ± 27 mg/dL	139 (119-258) mg/dL
		After tx	0.46 (0.29-1.87) mg/L	1602 ± 325 cm/s	136 ± 18/62 ± 9	72 ± 12	nm	47 ± 5 mg/dL	86 ± 16 mg/dL	121 (84- 167) mg/dL
		Before tx vs. after tx (*P* value)	<0.001	<0.01	ns	ns	—	<0.05	<0.001	<0.05
	Group B	Before tx	2.69 (1.69-7.09) mg/L	1876 ± 343 cm/s	136 ± 17/62 ± 10	69 ± 12	nm	43 ± 9 mg/dL	145 ± 29 mg/dL	141 (115-279) mg/dL
		After tx	0.44 (0.25-1.71) mg/L	1695 ± 338 cm/s	137 ± 20/63 ± 11	70 ± 11	nm	42 ± 8 mg/dL	117 ± 19 mg/dL	122 (88-175) mg/dL
		Before tx vs. after tx (*P* value).	<0.001	<0.05	ns	ns	—	ns	<0.01	<0.05

[23]	Group A	Before tx	1.78 ± 0.36 mg/L	1808 ± 328 cm/s	130 ± 14/78 ± 13	65 ± 9	245 ± 36 mg/dL	42 ± 6 mg/dL	157 ± 16 mg/dL	175 ± 39 mg/dL
		After tx	1.24 ± 0.29 mg/L	1653 ± 321 cm/s	131 ± 15/79 ± 15	66 ± 11	212 ± 28 mg/dL	40 ± 4 mg/dL	127 ± 15 mg/dL	163 ± 38 mg/dL
		Before tx vs. after tx (*P* value)	ns	<0.05	ns	ns	<0.05	ns	<0.05	<0.05
	Group B	Before tx	1.8 ± 0.41 mg/L	1806 ± 358 cm/s	132 ± 16/77 ± 11	68 ± 11	243 ± 38 mg/dL	41 ± 5 mg/dL	155 ± 18 mg/dL	173 ± 37 mg/dL
		After tx	1.82 ± 0.40 mg/L	2005 ± 429 cm/s	135 ± 20/78 ± 15	67 ± 10	214 ± 32 mg/dL	40 ± 4 mg/dL	129 ± 19 mg/dL	161 ± 39 mg/dL
		Before tx vs. after tx (*P* value)	<0.05 vs. final tx group A	<0.05 vs. before tx. <0.05 vs. final tx group A	For SBP: <0.05 vs. before tx. <0.05 vs. final tx group A	ns	<0.05	ns	<0.05	<0.05

[24]	FH		1.23 ± 1.66 mg/L	1257.3 ± 296.7 cm/s	115.2 ± 14.0 67.1 ± 10.4	nm	295.8 ± 71.7 mg/dL	57.9 ± 12.6 mg/dL	215.0 ± 64.2 mg/dL	129.5 ± 74.0 mg/dL
	Control		1.10 ± 1.08 mg/L	1196.4 ± 233.8 cm/s	107.2 ± 8.1/62.7 ± 7.6	nm	182.7 ± 21.8 mg/dL	52.3 ± 12.6 mg/dL	113.4 ± 22.9 mg/dL	107.5 ± 56.4 mg/dL
	FH vs. control (*P* value)		ns	ns	ns	nm	<0.001	ns	<0.001	ns

[25]	Men		3.67 ± 0.23 mg/dL	1429 ± 23.3 cm/s	125.6 ± 9.0/75.2 ± 6.8	74.6 ± 6.5	4.06 ± 0.22 mmol/L	1.75 ± 0.06 mmol/L	3.6 ± 0.9 mmol/L	1.93 ± 0.14 mmol/L
	Women		2.99 ± 0.25 mg/dL	1394 ± 26.2 cm/s	120.3 ± 10.5/70.6 ± 6.2	75.5 ± 5.8	3.35 ± 0.24 mmol/L	1.78 ± 0.06 mmol/L	3.4 ± 0.7 mmol/L	1.54 ± 0.17 mmol/L
	Men vs. women (*P* value)		ns	ns	<0.05 for both	ns	<0.05	ns	ns	<0.05

Abbreviation: CRP: C-reactive protein; FH: familial hypercholesterolemia; HC: hypercholesterolaemic; HDL-C: high-density lipoprotein cholesterol; hs-CRP: high-sensitivity C-reactive protein; LDL-C: low-density lipoprotein cholesterol; NL: normolipidemia; nm: not mentioned; ns: not significant; PWV: pulse wave velocity; SBP: systolic blood pressure; TC: total cholesterol; TG: triglyceride; tx: treatment.
